# Application of Immunoassay Technology in Food Inspection

**DOI:** 10.3390/foods12152923

**Published:** 2023-07-31

**Authors:** Peipei Li, Maojun Jin

**Affiliations:** Institute of Quality Standard and Testing Technology for Agro-Products, Chinese Academy of Agricultural Sciences, Beijing 100081, China; 18331090835@163.com

Food safety is as important as ever, and the safeguards implemented to inspect and reduce pesticides, veterinary drugs, toxins, pathogens, illegal additives, and other deleterious contaminants in our food supply has helped improve human health and increase the length and quality of our lives. Multiple countries and regions have established a series of food safety laws and national standards, which has driven the development of rapid detection methods for the measurement of hazards in food products. Benefiting from the merits of convenience, high efficiency, and on-site rapid detection, etc., immunoassays have achieved mainstream application in the rapid inspection field. As early as the mid-20th century, the first immunoassays were described for the measurement of insulin and thyroxine, respectively. Currently, hundreds of immunoassays have been established for scores of food hazards. Ouyang et al. [1] summarized the immunoassays used to detect β_2_-Agonists, including enzyme-linked immunosorbent assays (ELISA), lateral flow immunoassay (LFIA), fluorescence immunoassay, chemiluminescence immunoassay, etc. Generally, immunoassays are based on the principle of the specific recognition of antigens and antibodies. Antibodies serve as the basic recognition elements for immunological detection methods. Therefore, the design of hapten with high immunogenicity and preparation of antibodies with high specificity and affinity has become the most important part of immunoassays. In addition, improving the detection mode of immunoassays has become a research hotspot in recent years. Thus, this Special Issue provides some general methods used for hapten design and for the preparation of recognition elements, as well as an overview of immunoassay technology in food inspection.

The hapten design is key to the establishment of an immunoassay. The natural toxins α-solanine (SO) and α-chaconine (CHA) comprise approximately 95% of the total glycoalkaloid (GA) content in potatoes; ingestion of more than 1 mg of GAs per kg of body weight is toxic to humans. In order to simultaneously measure SO and CHA, Okada et al. [2] used solanidine, a chemical compound found in both SO and CHA, as the hapten for rabbit immunity. Two new polyclonal antibodies (pAbs) that bind to both SO and CHA were purified and obtained. Xu et al. [3] designed and synthesized a novel hapten of triazine herbicide atrazine (ATR), namely, 2-chloro-4-ethylamino-6-isopropylamino-1,3,5-triazine. This hapten maximally retains the characteristic structure of ATR and induces expected antibody response in the animal’s immune system. After cell fusion and screening, one monoclonal antibody (mAb) 9F5 was obtained with the isotype of IgG1. For the standard curve, the IC_50_ value of the 9F5 mAb was 1.678 μg/L with the working range (IC_20_–IC_80_) of 0.384 to 11.565 µg/L. In addition, this mAb showed high specificity to ATR and low cross-reactivity to other triazine herbicides. The prepared 9F5 mAb provided the core raw material for establishing an ATR immunoassay. In recent years, specific peptide ligands, such as peptidomimetic and anti-immunocomplex peptides, have been regarded as promising substitutes for chemical haptens. The peptidomimetic directly reacts with the antibody, while the anti-immunocomplex reacts with the immunocomplex between antibody and antigen. You et al. [4] identified thirty sequences of peptidomimetics and two sequences of anti-immunocomplex peptides for imidacloprid (IMI) from three phage display libraries, in which the anti-immunocomplex peptides were the first reported noncompetitive reagents for IMI. One peptidomimetic and one anti-immunocomplex peptide were utilized to develop competitive and non-competitive phage ELISA (P-ELISAs), with IC_50_ of 0.55 ng/mL and 0.35 ng/mL, respectively. Compared to chemical haptens, peptide ligands are prepared using biological expression. It is attractive to explore the peptide ligands to derive novel patterns of immunoassay.

In addition to the traditional pAbs and mAbs, more types of recognition elements have been discovered for immunoassay. Nanobodies, the smallest known functional antibody, have been used in the field of food safety. Qiu et al. [5] expressed a nanobody from a naive phage display library and used it as the capture antibody. Quantum dot (QB) was conjugated with a tnti-Cry2A pAb to serve as fluorescent probe. Then, a sensitive sandwich fluorescence-linked immunosorbent assay (QB-FLISA) using nanobody was established to determine the Cry2A toxin in cereal. The detection limit of this assay was 0.41 ng/mL, which had 19-times higher sensitivity than the traditional colorimetric ELISA. Known as “chemical antibodies”, aptamer has the advantages of simple preparation, stable properties, low immunogenicity, and easy modification compared with the antibody. Zhang et al. [6] used aptamer as recognition element and developed a competitive enzyme-linked aptamer assay (ELAA) kit to detect Lactoferrin (Lf) in dairy products. In the construction, the Lf aptamer was conjugated with horseradish peroxidase (HRP) as the recognition probe and an aptamer complementary strand was anchored onto the microplate as the capture probe. When TMB substrate appeared in the reaction system, the color shades were negatively correlated with the Lf concentrations in the sample. Under the optimization conditions, the aptamer-based ELAA kit achieved a good linear relationship (R^2^ = 0.9901) in the wide range of 25–500 nM with the detection limit of 14.01 nM and good specificity. In addition, Wang et al. [7] developed an aptamer-based surface-enhanced Raman spectroscopy (SERS) analysis for the anti-interference detection of Ochratoxin A (OTA). In this aptasensor, 4-[(Trimethylsilyl) ethynyl] aniline (TEAE) as an anti-interference Raman reporter, assembled on AuNPs with OTA-aptamer, served as SERS probes. Meanwhile, Fe_3_O_4_NPs, linked with complementary aptamer (cApts), were applied as capture probes. The specific binding of OTA to aptamer hindered the complementary binding of aptamer and cApt, resulting in a negative correlation between the Raman response at 1998 cm^−1^ and OTA levels. Under the optimum condition, the aptasensor presented a linear response for OTA detection in the range of 0.1–40 nM, with a low detection limit of 30 pM.

Many detection approaches have been developed to conduct qualitative/quantitative analysis of hazards in food samples. Zhu et al. [8] established an indirect competitive chemiluminescence enzyme-linked immunoassay (Cl-ELISA) to detect acetamiprid (ACE) in the Chinese cabbage and cucumber. The LOD of this assay was 1.26 μg/kg, meeting the MRL requirements of ACE in vegetables. Subsequently, Zhai et al. [9] compared Cl-ELISA with colorimetric ELISA (CO-ELISA) for IMI detection in vegetables. The results indicated that Cl-ELISA showed high sensitivity and a rapid detection time, saving costs (antigen and antibody concentrations) and serving as a more efficient model for the rapid detection of IMI residue, which provides a theoretical basis for selecting an optical detection assay. The improvement of the sensitivity of immunoassay has always been the focus of researchers. Hendrickson et al. [10] developed a cascade-enhanced LFIA to detect okadaic acid (OA) in seawater, fish, and seafood. In the case of the cascade-enhanced LFIA, the anti-OA antibodies specifically bound to the OA-BSA on the T line of the strip, followed by several (at least two) cycles of successive passing gold-labeled goat anti-mouse antibodies (GAMI–AuNPs) and free donkey anti-goat antibodies (DAGI) that were not specific to OA. As a result, branched aggregates formed on the T line with a (GAMI-AuNPs-DAGI) × *n* structure, where *n* is the number of GAMI-AuNPs/DAGI passing cycles. The LOD of the cascade-enhanced LFIA enabled the detection of OA at 30 pg/mL, which was two to seven times higher than the LOD of the LFIA without amplification. Immunomagnetic beads (IMBs) have been widely used to capture and isolate target pathogens from complex food samples. Kang et al. [11] proposed a pH-regulated strategy to orient the antibody on the surface of MBs. This study revealed that the positively charged ε-NH_2_ group of lysine on the Fc relative to the uncharged amino terminus on Fab was preferentially adsorbed on the surface of MBs with a negatively charged group at pH 8.0, resulting in the antigen-binding sites of the antibody being fully exposed. This strategy can efficiently capture and isolate pathogenic microorganisms from complex food matrices at low cost, thereby improving the specificity and sensitivity combined with immunoassay.

In summary, this Special Issue provides insights into the recent application of immunoassay technology in food inspection. We sincerely hope that readers will find this Special Issue informative and interesting.
